# Improvement of amperometric transducer selectivity using nanosized phenylenediamine films

**DOI:** 10.1186/s11671-017-2353-9

**Published:** 2017-11-14

**Authors:** O. V. Soldatkina, I. S. Kucherenko, V. M. Pyeshkova, S. A. Alekseev, O. O. Soldatkin, S. V. Dzyadevych

**Affiliations:** 1 Taras Shevchenko National University of Kyiv, Volodymyrska Street 64, Kyiv, 01601 Ukraine; 2 Laboratory of Biomolecular Electronics, Institute of Molecular Biology and Genetics, National Academy of Sciences of Ukraine, Zabolotnogo Street 150, Kyiv, 03148 Ukraine

**Keywords:** Amperometric sensor, Permselective membrane, Phenylenediamine, Electroactive substance, Ascorbic acid, Biosensor

## Abstract

In this work, we studied the conditions of deposition of a semipermeable polyphenylenediamine (PPD)-based membrane on amperometric disk platinum electrodes. Restricting an access of interfering substances to the electrode surface, the membrane prevents their impact on the sensor operation. Two methods of membrane deposition by electropolymerization were compared—at varying potential (cyclic voltammetry) and at constant potential. The cyclic voltammetry was shown to be easier in performing and providing better properties of the membrane. The dependence of PPD membrane effectiveness on the number of cyclic voltammograms and phenylenediamine concentration was analyzed. It was shown that the impact of interfering substances (ascorbic acid, dopamine, cysteine, uric acid) on sensor operation could be completely avoided using three cyclic voltammograms in 30 mM phenylenediamine. On the other hand, when working with diluted samples, i.e., at lower concentrations of electroactive substances, it is reasonable to decrease the phenylenediamine concentration to 5 mM, which would result in a higher sensitivity of transducers to hydrogen peroxide due to a thinner PPD layer. The PPD membrane was tested during continuous operation and at 8-day storage and turned out to be efficient in sensor and biosensors.

## Background

Biosensors are novel analytical devices; the usage of which is an alternative to chromatography, spectroscopy, and colorimetry. Biosensors are much cheaper and easier to use than these traditional methods, however, often inferior to them by the analytical characteristics. At present, the research in the field of biosensorics is in active progress [1].

According to the classical definition of the International association of researchers in fundamental and applied chemistry, a biosensor is an integrated device based on receptor and transducer, which is able to provide quantitative or semi-quantitative analysis using biological recognition element [2]. By the type of transducer, biosensors are classified into several groups (electrochemical, optical, piezoelectric, etc.), among which electrochemical biosensors are one of the largest groups and in turn are divided into amperometric, potentiometric, conductometric, and impedimetric [3].

One of important analytical characteristics of biosensors is their selectivity, i.e., an ability to identify the target compound only. The biosensor selectivity is determined by selectivity of biological material and selectivity of transducer. Basically, enzymes and antibodies used in electrochemical biosensors as biomaterial are very selective, whereas electrodes, which serve as transducers, are rather nonselective. The biosensor selectivity is of particular importance when working with real biological fluids or other complex samples; therefore, its investigation is a necessary stage in the development of biosensors.

In blood serum, urine, cerebrospinal fluid, etc., there are interfering substances that can undergo chemical reactions on the surface of transducers, thus causing false results in biosensor measurement of the target substance. The main interferents in biological samples are ascorbic acid, cysteine, homocysteine, uric acid, dopamine, glutathione, etc. Their concentrations in human blood serum are shown in Table 1.

**Table 1:** Concentration of electroactive substances in human blood serum

Substance	Concentration in serum	Reference
Ascorbic acid	0.4–2.0 mg/dL = 23–114 μM	[16]
	1.8–17.9 mg/L = 10.2–101 μM	[17]
Cysteine	255.9–317.3 μM	[18]
Total homocysteine	6.8–17.0 μM	[18]
Uric acid	120–450 μM	[19, 20]
	208–428 μM (males) 155–357 μM (females)	[21]
Dopamine	0.01–1 μM	[22]
	1 nM (healthy people) 0.9–6 μM (patients with Parkinson’s disease treated with l-dopa)	[23]
Total glutathione	5.8 ± 0.9 μM	[18]
Reduced glutathione	1.6 ± 0.3 μM	[18]

There are two main approaches to prevent oxidation of interfering substances on the electrode surface—a decrease of working potential by introduction of additional substances into the bioselective membrane or deposition of additional semipermeable membranes, which allows selective access of the target substance to the electrode surface [4]. The deposition of semipermeable membranes is methodologically simpler and slightly affects the biosensor functioning.

In biosensors, hydrogen peroxide oxidizes or reduces on the electrode and thus the biosensor signal is generated. Therefore, an actual issue is the development of nanoporous films, which are permeable for hydrogen peroxide and prevent the penetration of other substances. Among these membranes, the polymer films based on phenylenediamine (PD) attract considerable attention [5]. Polyphenylenediamine (PPD)-based membrane has nanopores; their size is sufficient for penetrating low molecular weight compounds, including hydrogen peroxide, through membrane to the electrode surface. On the other hand, the membrane does not allow passing through or oxidation of larger substances like ascorbic acid or dopamine. Thus, the membrane improves selectivity to hydrogen peroxide, which, in turn, increases the biosensor accuracy. In several works, different PD isomers and methods for PD polymerization were studied. Particularly, PPD membranes were formed on ruthenium-coated carbon fiber microelectrodes by electrodeposition at a constant potential (+ 0.7 V) when creating biosensors based on glucose oxidase, lactate oxidase, and glutamate oxidase [6]. Three PD isomers were tested; the results with meta-isomer were the best. Because some sensitivity to ascorbic acid still remained, ascorbate oxidase was added to eliminate it completely. In [7], the authors studied PPD membranes deposited onto Pt–Ir cylinders by CV or constant potential amperometry. Sensitivity to ascorbic acid notably decreased with the membranes based on meta- and ortho-isomers oxidized at a constant potential whereas sensitivity to hydrogen peroxide decreased by 10% only. The results obtained with PPD membranes deposited onto palladium disk electrodes were quite different [8]. Electrodeposition of *m*-PD by CV caused the formation of films with threefold higher hydrogen peroxide permeability compared with *m*-PD oxidation at a constant potential. Thus, *m*-PD was shown to be preferable among all isomers. The recently reported hydrogen peroxide sensor using CV-deposited *o*-PD film with Au nanoparticles [9] demonstrated good avoidance of interfering effects. Generally, it can be concluded that *m*-PD is superior to others for all electrodes, whereas the procedure of PD polymerization should be optimized in each particular case. Additionally, PD-based membranes can be also used in sensors without the biological element. As was recently shown, bovine serum albumin could be detected by a sensor based on conjugated copolymers of PD and other aromatic compounds (quenching of the protein fluorescence after binding with the copolymer was observed) [10].

Thus, the aim of the present work was to compare different methods of *m*-phenylenediamine deposition and select an optimal procedure of PPD formation on the platinum disk electrodes.

## Methods

### Materials

Ascorbic acid, cysteine, uric acid, dopamine, hydrogen peroxide, *m*-phenylenediamine, and HEPES were purchased from Sigma-Aldrich Chemie (USA). All other chemicals were of p.a. grade.

The samples of human blood serum were obtained from Kyiv Municipal Scientific and Practical Center of Nephrology and Hemodialysis (Ukraine).

### Design of Amperometric Transducers

In this work, self-made platinum disk electrodes served as amperometric transducers. Platinum wire 0.4 mm in diameter and 3 mm long was sealed at one end of a glass capillary with an outer diameter of 3.5 mm. An open end of the wire was the working surface of transducer. An inner end of the platinum wire was soldered by Wood’s alloy to one end of a silver wire inside the capillary; its another end was connected to a potentiostat. The electrodes were used repeatedly; before usage, their working surface was treated with HCl for 30 s, washed with ethanol, and grinded by abrasive paper P1500 PS 8A.

### Methods of Measurement

The UV-vis absorption spectra of the samples were measured on Thermo Evolution 600 spectrometer in the 200–900-nm wavelengths range in diffuse-reflectance mode using an integration sphere. The Spectralon diffuse reflectance standard and platinum disk were used as blank samples for *m*-phenylenediamine powder and PPD layer on the surface of Pt electrode respectively.

For electrochemical measurements, working electrodes were placed in a classical electrochemical cell with an auxiliary (platinum wire) and a reference (Ag/AgCl in saturated KCl) electrodes connected to the PalmSens potentiostat (Palm Instruments BV, Netherlands). Usage of the eight-channel multiplexer (from the same producer), connected to the potentiostat, allowed simultaneous monitoring of signals from eight electrodes; however, in our work, we usually used three electrodes because of small size of the working cell.

The chronoamperometric measurements (“amperometric detection” technique) were carried out at room temperature in an open 3-mL glass cell with permanent stirring by a magnetic stirrer and at constant potential of 0.6 V versus Ag/AgCl reference electrode. Ten millimolars of HEPES, pH 7.4, was used as a working buffer in all experiments. The substrate concentrations in the working cell were obtained by addition of aliquots of stock solutions (50 mM hydrogen peroxide, 20 mM ascorbic acid, 3 mM cysteine, 4.5 mM uric acid, 2.1 mM dopamine). New solutions were prepared just before the experiment. All electroactive substances except uric acid were dissolved in the working buffer; uric acid due to its small solubility was dissolved in distilled water with 5 mM NaBrO_3_. Phenylenediamine was dissolved in 40 mM phosphate buffer, pH 7.4.

Cyclic voltammetry was carried out in the same measuring cell without stirring. Start potential was 0 V, final potential +0.9 V, scan rate (rate of potential change) 20 mV/s, and step of potential change 5 mV.

All experiments were carried out in three repetitions. Data in the tables and figures represent a mean value of experiments ± standard deviation, computated by OriginLab OriginPro 8.5 program.

## Results and Discussion

To confirm the reasons of deposition of additional membrane on amperometric transducer for improvement of its selectivity to hydrogen peroxide, it was necessary to verify sensitivity and selectivity of this transducer regarding possible interferents.

Biosensors can be employed for measurements in both undiluted and diluted samples. The option of dilution depends on the concentration of substance to be analyzed and the biosensor sensitivity: if the biosensor can identify the target substance in concentrations, which are dozens of times lower than those actual in the samples, the latter should be diluted to decrease the content of interferents and thus improve the array accuracy. Additionally, it allows dozens-fold decrease of the substrate volume required for measurements.

Sometimes the concentration of analyzed substance is too low, or dilution is undesirable for technical reasons. For better understanding of the function of amperometric transducer, electroactive materials of three concentrations were used: (1) relevant for blood serum, (2) 20-fold lower than (1), i.e. 20-fold dilution, and (3) 100-fold dilution. The biosensor responses to electroactive substances of three concentrations were received using the bare transducer before the PPD film deposition, and transducer sensitivity was calculated (Table 2). As seen, the sensitivity to dopamine and ascorbic acid was the highest, to cysteine —the lowest. However, ascorbic and uric acids can be considered as main interferents due to their essentially higher concentrations in biological samples. The transducer responses to these substances are an order of magnitude larger than the response to the target substance, hydrogen peroxide. Therefore, the bare transducer is unsuitable for measurements in biological samples due to the pronounced impact of interferents. On the other hand, after 100-fold dilution, the responses to cysteine and dopamine became negligible, and response to all interferents in total was only about 20% of the response to hydrogen peroxide, which means that in some cases, the sensor can be used even without additional modifications.

**Table 2:** Responses and sensitivity of platinum disk transducer to electroactive materials. Responses are received in 10 mM HEPES buffer, pH 7.4

Analyzed substance	Undiluted sample	20-fold dilution of the sample		100-fold dilution of the sample		Mean sensitivity of transducer, nA/mM
	Transducer response, nA	Substance concentration in working cell, μM	Transducer response, nA	Substance concentration in working cell, μM	Transducer response, nA	
H_2_O_2_	4.5 ± 1.5	50	4.6 ± 1.4	50	4.2 ± 1.6	90 ± 30
Ascorbic acid	52.5 ± 25.1	6	2.7 ± 1.1	1.2	0.4 ± 0.2	438 ± 209
Cysteine	9.0 ± 5.7	15	0.5 ± 0.3	3	0.1 ± 0.1	30 ± 19
Uric acid	62.4 ± 31.2	22.5	3.0 ± 1.4	4.5	0.6 ± 0.2	139 ± 70
Dopamine	2.7 ± 0.5	0.3	0.1 ± 0.1	0.06	0	450 ± 83

Currently, there is only fragmented information regarding the methods of PPD membrane deposition on transducers. Therefore, at the next stage of work, it was evaluated which method of two most common and promising is more feasible.

**Fig. 1:**
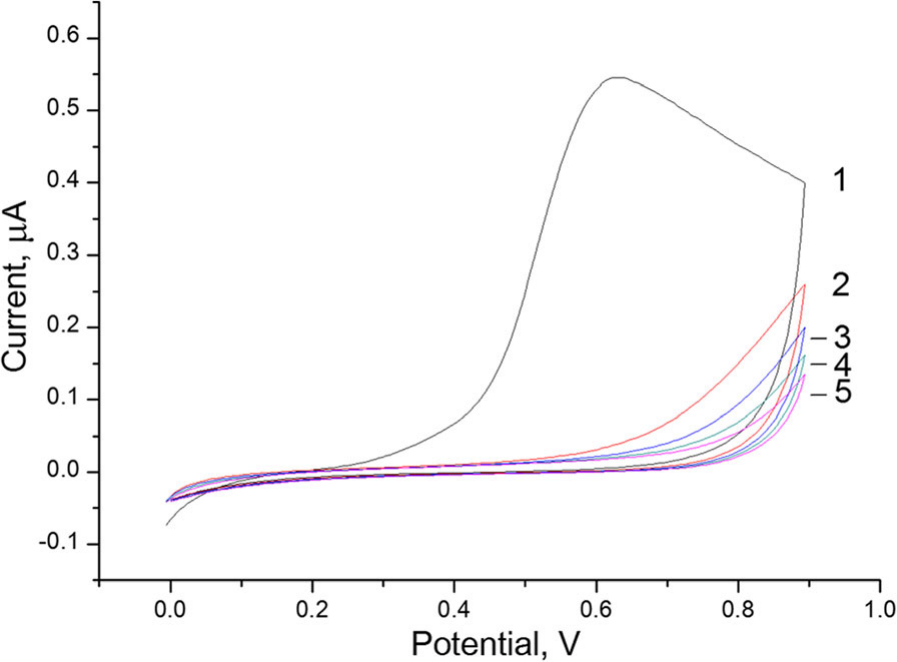
Cyclic voltammograms obtained at phenylenediamine electropolymerization on transducer surface

In the first method, PPD membranes were deposited on the surface of platinum disk electrode via electropolymerization of molecules of monomer phenylenediamine using varying potential (cyclic voltammetry). The transducers with reference and auxiliary electrodes were placed in a working cell with phenylenediamine solution, and a number of cyclic voltammograms were obtained [7]. An example of the experiment is shown in Fig. 1. During the first cyclic voltammogram (CVA), a significant increase in the current was observed at the potential ranging from 0.5 to 0.9 V because of phenylenediamine oxidation. During the second and subsequent CVAs, the current significantly decreased, indicating a lower speed of electropolymerization. However, as the following experiments showed, the formation of PPD membrane lasted over all CV.

The second method of PPD membrane deposition consists in phenylenediamine oxidation at a constant potential of + 0.7 V over fixed time (40 min) [11]. Comparison of transducer responses when using both methods of deposition is shown in Table 3. Hereafter, the responses to the electroactive substances without the PPD membrane were taken as 100%. The membranes, deposited by both methods, impeded interferents quite effectively—only a weak sensitivity to cysteine was observed. On the other hand, the transducer sensitivity to hydrogen peroxide after voltammetry increased by 2.6 times. This can be explained by the platinum electro-activation during voltammetry, but not by the effect of PPD membrane. Such increase in sensitivity to hydrogen peroxide was observed also after obtaining cyclic voltammograms in phosphate buffer without phenylenediamine. After the membrane deposition at a constant potential, no electro-activation was revealed and the responses to hydrogen peroxide did not change. Thus, the use of cyclic voltammetry was revealed to be preferable for three reasons—less time for one deposition (20 vs. 40 min), more efficient hindering of cysteine, and increasing responses to hydrogen peroxide.

**Table 3:** Comparison of standard methods of deposition of PPD membrane

Analyzed substance and its concentration	10 CVAs in 5 mM phenylenediamine		40 min at a constant potential of 0.7 V in 100 mM phenylenediamine	
	Response, nA	% of response without membrane	Response, nA	% of response without membrane
H_2_O_2_, 50 μM	12.0 ± 1.4	267.3 ± 26.0	4.5 ± 1.3	99.3 ± 29.7
Ascorbic acid, 120 μM	< 0.1	0	< 0.1	0
Cysteine, 300 μM	0.2 ± 0.2	1.9 ± 1.9	0.6 ± 0.4	6.3 ± 3.8
Uric acid, 450 μM	< 0.1	0	< 0.1	0
Dopamine, 6 μM	< 0.1	0	< 0.1	0

However, cyclic voltammetry has one disadvantage—voltammograms may be simultaneously obtained on one electrode only (even using multiplexer), whereas membrane deposition at a constant potential allows simultaneous connection of 8–16 working electrodes (depending on the multiplexer type). Therefore, further work should be focused on optimization of the conditions of cyclic voltammograms to decrease the time of transducer pretreatment.

PD electropolymerization by CV and constant potential amperometry is supposed to occur by different pathways through quite complicated mechanism [12]. Thus, CV involves high applied potential, which leads to the generation of less conjugated oligomers of PD. For this reason, it is supposed that at PD polymerization by CV, the pores are larger, and the PPD layer permeability is higher if compared to the polymerization at a constant potential [8, 13]. However, as noted in the “Background” section, different authors came to contradictory conclusions about the preferential method of PD deposition, and in many cases, CV gave good results. In our opinion, both CV and constant potential amperometry can provide the generation of PPD membranes with good permselective properties, and optimization is necessary for each particular case.

**Fig. 2:**
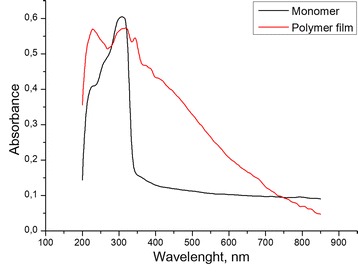
UV-vis diffuse reflectance spectra of *m*-phenylenediamine and PPD membrane, formed on the Pt electrode

The PPD membrane was clearly visible on the electrode surface as a uniform transparent golden-brown film. To confirm that it is PPD indeed, the PD polymerization was additionally confirmed by spectroscopy. The UV-vis diffuse reflectance spectrum of the film (Fig. 2) demonstrates intense absorbance bands at 222 and 315 nm, similar to the bands of the monomer and related to the electron transitions in the aromatic rings [14], whereas the featureless absorbance at 400–800 nm, constantly decreasing with the wavelength relates to *π* − π* electron transitions in highly conjugated aromatic system of conductive PPD polymer.

For better interpretation of the obtained results, it would be useful to estimate the size of pores in the PPD membranes produced by different methods. However, direct determination of the pore size in PPD membrane is almost impossible, since the membrane consists of multiple layers of PD and pores in bottom layers can have different size. Killoran and O’Neill determined that the thickness of effective membrane from *m*-PD was 15 nm, and cross-sectional area of one oligomeric polymer strand estimated by del Valle et al. was 1 nm [7, 15]. Thus, the PPD membrane contains roughly 15 layers of polymer. Since the PPD membrane has hydrophobic and isolating properties, the membrane should have perforating nanopores that stretch to the electrode surface and allow by-pass of hydrogen peroxide molecules, otherwise H_2_O_2_ cannot be oxidized and generate amperometric signal. The pores are definitely not uniform and the minimal diameter of pores should be less than 1 nm in order to reject electroactive molecules, and thus it is quite difficult to analyze the pores even with electron or atomic force microscopes. For these technical reasons, it is much easier to estimate effectiveness of the PPD membrane by evaluating the membrane permeability for different molecules. Such indirect approach is widespread and allows comparison of practical characteristics of different membranes.

**Fig. 3:**
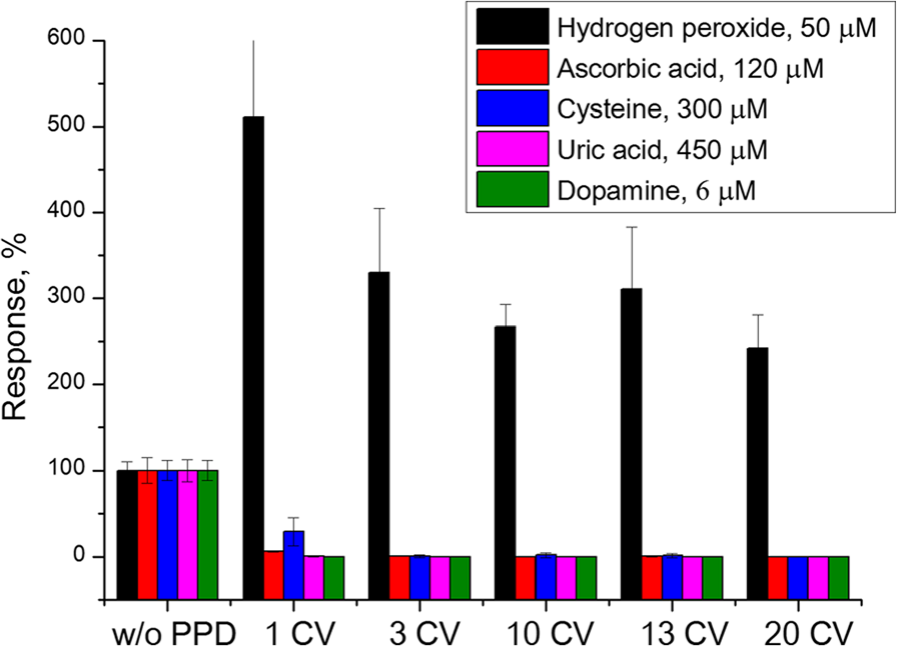
Efficiency of PPD membranes deposited using different number of CVAs

The effectiveness of PPD membranes deposited using various number of cyclic voltammograms was tested (Fig. 3).

**Fig. 4:**
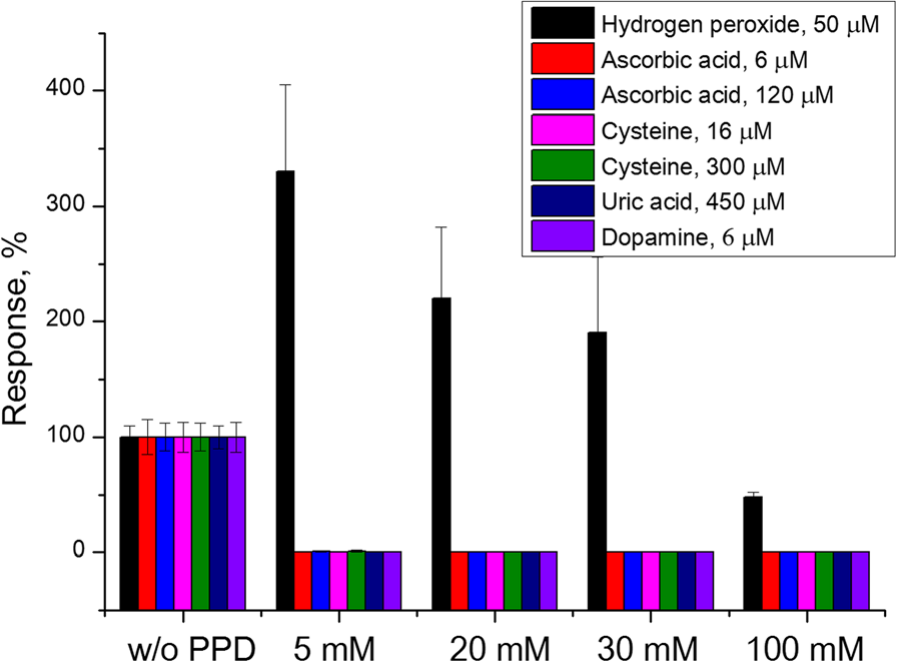
Efficiency of PPD membranes deposited at different phenylenediamine concentrations

PPD membrane deposited by one CVA was obviously insufficient to eliminate the influence of interferents. However, here, the effect of platinum electroactivation was the strongest. At further increase of the voltammograms number, the responses to interferents decreased, but at the same time, the transducer sensitivity to hydrogen peroxide also decreased, probably because of too thick PPD layer, which impedes the substance diffusion. Three CVAs were enough for complete disappearance of responses to dopamine and uric acid, and significant decrease in responses to ascorbic acid and cysteine. Therefore, three CVAs were taken for optimal, and phenylenediamine concentration was increased up to complete elimination of the interferent impact (Fig. 4).

Notably, the use of 5 mM phenylenediamine was sufficient to eliminate the responses to interferents of small concentrations remaining after sample dilution, but deficient to work with undiluted samples. An increase of phenylenediamine concentration to 20 mM and three CVAs turned out to be sufficient for complete elimination of the cysteine impact and a decrease of responses to ascorbic acid to the lowest level (0.1% of the response to ascorbic acid without PPD membrane). The use of higher (up to 100 mM) phenylenediamine concentration resulted in twofold decrease of the transducer sensitivity to hydrogen peroxide, probably, because of too thick PPD layer. Thus, the deposition of PPD membrane using three CVAs in 30 mM phenylenediamine is an optimal procedure. As one voltammogram lasted about 2 min, the membrane deposition on one sensor took 6 min.

**Fig. 5:**
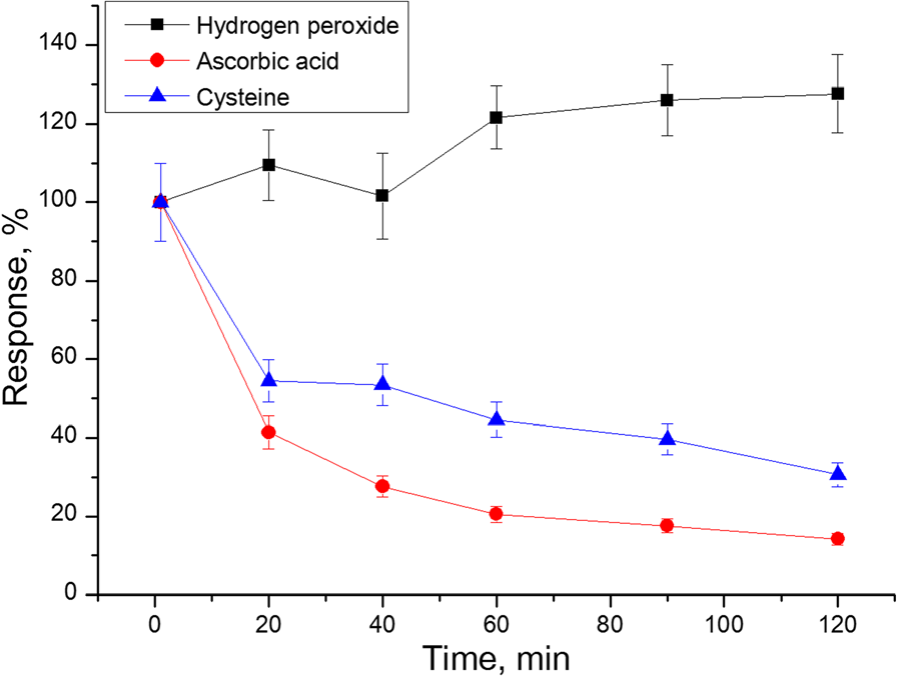
Stability of the PPD membrane during 2 hours. The responses to three substances were normalized to the initial response to the corresponding substance after PPD deposition

Next, stability of the PPD membrane was studied. After deposition of the PPD membranes, the sensors were placed in the working buffer for 2 h, and responses to hydrogen peroxide, ascorbic acid, and cysteine were measured to evaluate the changes in membrane permselectivity (Fig. 5). It was found that the responses to hydrogen peroxide slightly increased during the work, whereas small responses to interferents became even smaller. Probably, it happened because ascorbic acid and cysteine gradually clogged few large pores in the PPD layer. This experiment demonstrated that the PPD membrane can be used with no significant loss in its selectivity to hydrogen peroxide during at least 2 h.

**Fig. 6:**
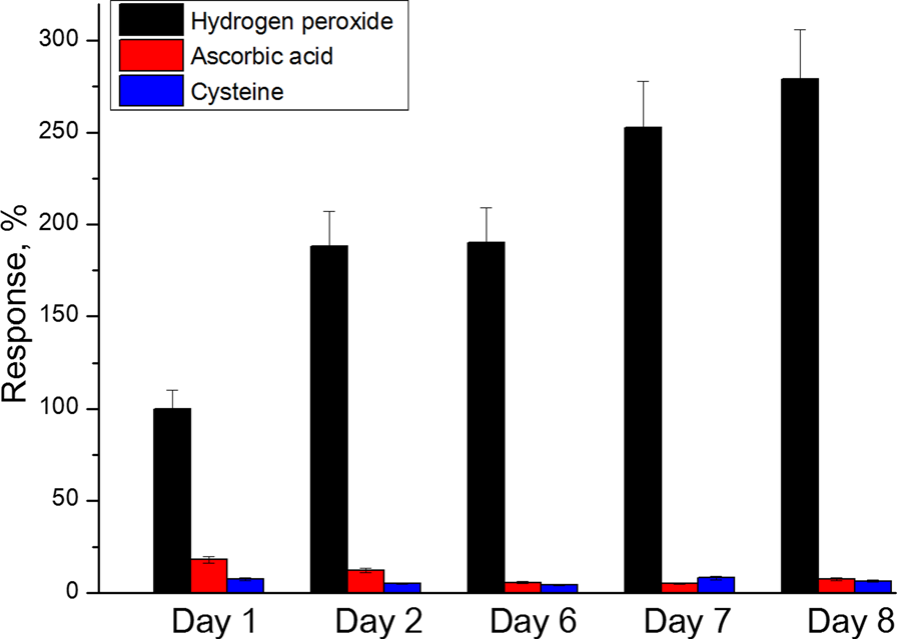
Storage stability of the PPD membrane. The responses were normalized to response to H_2_O_2_ at the first day

Storage stability of the PPD membrane was investigated. The sensors with deposited PPD membranes were stored dry during 8 days at − 18 °C; periodically, the sensors were unfrozen and responses to hydrogen peroxide, ascorbic acid, and cysteine were measured (Fig. 6). Over this period, the sensitivity of sensors to hydrogen peroxide increased by 2.5 times; the sensitivity to ascorbic acid and cysteine did not change. This effect can be explained by slow swelling of the PPD membrane that led to an improvement of hydrogen peroxide diffusion through the PPD layer.

Finally, the PPD membrane effectiveness was validated at analysis of real biological samples. The transducers with no membranes demonstrated weak signals after addition of blood serum to the working cell due to the presence of electroactive compounds. However, after deposition of the membrane, no responses were obtained. Similar results were obtained with the lysate of neurons. These experiments demonstrate that the developed method of PPD deposition on platinum disk electrodes is effective and the modified transducers can be used for work with complex biological samples.

It is useful to compare the method for PPD deposition developed in this work with the methods reported earlier (Table 4).

**Table 4:** Comparison of the developed method of deposition of PPD membrane with previously reported methods

Optimal method of PD polymerization	Type of electrode	Optimal isomer of PD	Duration of the electrode preparation	Effectiveness of blocking properties	Storage stability	Biosensor application	Ref.
CV (from − 0.3 V to +0.9 V vs. Ag/AgCl)	Pd disk	*m*-PD	48 min	Full blocking of AA, cys, acetaminophen. Small interference from UA	N/A	N/A	[8]
Constant potential (+0.7 V vs. Ag/AgCl)	Ru-covered carbon fiber ME	*m*-PD	45 min	Full blocking of UA, cys, acetaminophen, DA. Small interference from AA—addition of ascorbate oxidase to remove it	> 2 days	Glucose, lactate and glutamate biosensors, in vivo analysis in rat brain	[6]
Constant potential (+0.7 V vs. SCE)	Pt–Ir cylinders ME	*o*-PD	15 min	Full blocking of AA	> 7 days	N/A	[7]
CV (from −0.25 V to +0.9 V vs. SCE)	Glassy carbon electrode	*o*-PD	15 min	Full blocking of AA, UA and DA	> 15 days	N/A, H_2_O_2_ detection in human serum samples	[9]
Constant potential (+0.7 V vs. Ag/AgCl)	Pt cylinders ME	*o*-PD	15 min	Full blocking of AA	N/A	N/A	[13]
CV (from 0 V to +0.9 V vs. Ag/AgCl)	Pt disk	*m*-PD	6 min	Full blocking of AA, cys, UA, DA	> 8 days	Human serum samples and cell lysate	This work

*ME* microelectrode, *AA* ascorbic acid, *cys* cysteine, *DA* dopamine, *UA* uric acid, *N/A* not applicable

As seen, the presented method is the quickest one and blocking properties of the obtained membrane are better or at least not worse than those of other PPD membranes.

## Conclusions

We investigated the conditions of deposition of a semipermeable polyphenylenediamine-based membrane aimed at decreasing an impact of interfering substances on the biosensor operation. It was shown that phenylenediamine electropolymerization by cyclic voltammetry was easier and provided better properties of the membrane if compared to electropolymerization at constant potential. The dependence of PPD membrane effectiveness on the number of cyclic voltammograms and phenylenediamine concentration was investigated. It was shown that the impact of interfering substances on the sensor operation can be completely eliminated by using three cyclic voltammograms in 30 mM phenylenediamine. On the other hand, when working with diluted samples, i.e., lower concentrations of electroactive substances, it is reasonable to decrease the phenylenediamine concentration to 5 mM, which would result in higher transducer sensitivity to hydrogen peroxide due to a thinner PPD layer. The PPD membrane can be used with no significant loss in its selectivity to hydrogen peroxide during at least 2 h of continuous operation and can be stored at least 8 days. It was shown that the transducer with the PPD membrane is not sensitive to the electroactive substances present in biological samples and can be used for the biosensor creation.
